# Determinants of Financial Empowerment Among Women in Saudi Arabia

**DOI:** 10.3389/fpsyg.2021.747255

**Published:** 2021-10-15

**Authors:** Murad Ali, Imran Ali, Saeed Badghish, Yasir A. Soomro

**Affiliations:** ^1^Faculty of Economics and Administration, King Abdulaziz University, Jeddah, Saudi Arabia; ^2^Newcastle Business School, Northumbria University, Newcastle upon Tyne, United Kingdom

**Keywords:** financial literacy, financial socialization, financial coping behavior, Saudi women, financial empowerment, financial self-efficacy

## Abstract

Increasing women’s financial empowerment is important as they experience a lack of control over economic resources as compared to men. Although plenty of research evidence is available on the determinants of financial empowerment among women in developed countries, there is less known in the context of a traditionally male-dominated society like Saudi Arabia. The current study proposes a conceptual model that examines the role of financial literacy and financial socialization, in the development of financial self-efficacy, financial coping behaviors, and financial empowerment among Saudi women using social cognitive theory (SCT). Data are collected through a baseline survey from a sample of 1,368 women respondents who belong to the different segments of society ranging from female university students to women in the household to women working in different sectors in Saudi Arabia. We employed partial least squares (PLS) path modeling techniques using SmartPLS to test the hypotheses proposed in this study. The study found a significantly positive association between financial literacy, financial coping behavior, and financial well-being. Financial socialization is also significantly related to financial self-efficacy and financial empowerment. We also found the positive role of financial self-efficacy and financial coping behaviors in the development of financial empowerment. The practical implication of this study includes the provision of financial literacy education/training to Saudi women and increasing their financial socialization to improve their financial well-being.

## Introduction

Financial well-being has become an important topic during recent years. Many scholars have identified the gender gaps in financial well-being, its antecedents, and outcomes (Goncalves et al., 2021). Financial literacy has emerged as an important determinant of financial well-being and financial empowerment. Financial literacy is becoming even more important as the financial world is enduring more complexities. Therefore, a better understanding of financial concepts and financial products has become inevitable to develop skills for making sound financial decisions to improve financial well-being and empowerment (Philippas and Avdoulas, 2020).

Financial literacy refers to “the perception of being able to sustain current and anticipated desired living standards and financial freedom” (Brüggen et al., 2017, p. 229). Financial literacy is “the ability to use knowledge and skills to manage financial resources effectively for a lifetime of financial well-being.” Research suggests that communities with better financial literacy live prosperous and happy lives. Goncalves et al. (2021) highlight the wider gender gap of financial literacy among men and women. Financial literacy is very important for women to promote their economic empowerment. As the UN believes, “*If you educate a man, you educate one person, if you educate a woman, you educate a nation*.*”* Therefore, improving financial literacy is not only important for themselves but also for the generations to come.

Apart from gender differences in financial literacy, there is a significant financial literacy gap among people across different regions (Lusardi and Mitchell, 2011). There is clearly a wide gap of financial literacy level between developed and less developed countries (Lusardi and Mitchell, 2011). For the Arab region, according to the World Bank’s (2016) report, historically financial inclusion in the Arab world has been low as compared to other countries, and women are more vulnerable as compared to men in making financial decisions related to saving and choosing financial products. Another important study by Hasler and Lusardi (2017) at the Global Financial Literacy Excellence Center (GFLEC) for the G20 countries reports that Saudi Arabia has one of the lowest financial literacies (women around 29% and men around 34%) among G20 countries, whereas the United Kingdom has the highest financial literacy rate among women (around 72%), and Canada has the highest financial literacy rate among men (around 78%) (S&P Global FinLit Survey and Global Findex Database, 2014). In another indicator, the percentage of Saudi women borrowing from financial institutions is around 6%, whereas it is around 24% in Australia and 25% in Canada. This shows that although Saudi Arabia has generally promising economic indicators, they have a quite low score in terms of financial literacy with women lower than men. Financial literacy can help women to gain confidence in and control over their financial decisions. Consequently, these individuals are better able to control their social standing. It follows that social empowerment, though often overlooked, should be an integral part of any financial literacy initiative.

The current study employs social cognitive theory (SCT), which was proposed by Bandura (1986), and postulates the triadic reciprocity and the interrelation between individual, ecological, and attitude aspects. Martin et al.(2014, p. 2) hold that “SCT estimates the ability of an individual to engage in a targeted behavior, based on internal and external parameters and their interrelationships.” In this study, we assume the triadic interrelationship between (i) external factors, i.e., financial socialization factors, and institutions that provide financial literacy to (ii) Saudi women (individual factors) to (iii) shape their financial management behavior that promotes financial empowerment among Saudi women. Thomas and Gupta (2021) also employed SCT to examine the association between financial literacy and financial well-being.

The study offers an important contribution to the body of knowledge and practice by responding to the research call by Lusardi and Tufano (2009); Hasler and Lusardi (2017), Philippas and Avdoulas (2020); Goncalves et al. (2021), and Thomas and Gupta (2021). It offers a theoretical framework that examines the role of financial literacy and financial socialization in improving financial self-efficacy, financial coping behavior, and financial empowerment among Saudi women. This study offers a unique perspective to examine the recent public policy initiatives undertaken by the Saudi government to improve women’s financial literacy, their financial management skills, and financial empowerment so that they can play an active role in the socioeconomic development of the Kingdom as envisaged in the Vision 2030. The following sections describe the theoretical background and hypotheses proposed in this study, the research methods employed, data analysis, and the discussion and conclusion of this research.

### Theoretical Background and Development of Hypotheses

#### Financial Literacy, Financial Self-Efficacy, Financial Coping Behavior, and Financial Empowerment

The fundamental goal of financial literacy is to create financial empowerment among its targeted communities. Financial empowerment may be understood as the capacity of individuals to access and engage in financial growth processes, and be in a position to negotiate a fairer distribution of the benefits (Eyben et al., 2008). This enables them to think beyond day-to-day survival and exercise greater control over their resources and financial decisions. Financial literacy plays a central role in supplying individuals with knowledge, skills, and confidence, which are vital to achieve financial empowerment (Postmus, 2011a).

The greatest need for financial empowerment exists among women and minority groups that tend to be, for various reasons, sidelined from financial responsibilities and decision-making. One of the most detrimental, but perhaps overlooked, impacts of this on the well-being of women is economic abuse by their partners, which involves a range of tactics employed by the abuser to control a woman’s ability to acquire, use, and maintain economic resources with the intention of threatening her economic security and undermining her economic independence (Postmus, 2011a). Economic dependence is also one of the main reasons reported by intimate partner survivors (IPVs), especially poor women lacking the resources to live independently, for returning to their abusive partners (Sanders and Schnabel, 2006; Postmus, 2011a). To combat economic abuse, financial literacy initiatives aimed at developing women’s knowledge, skills, and confidence in personal finance often serve as the vehicle for enabling financial empowerment (Postmus, 2011b). For example, the Intervention with Microfinance Study for AIDS and Gender Equality (IMAGE) initiative halved the level of IPV in South Africa (Vyas and Watts, 2009). Similarly, the Allstate Foundation’s Moving Ahead through Financial Management curriculum was found to have significantly improved survivors’ financial empowerment in terms of financial literacy, economic self-efficacy, and economic self-sufficiency (Postmus, 2011b). This would, consequently, improve their personal safety, financial security, and long-term economic stability.

By enabling financial empowerment, financial literacy also supports the broader goal of gender equality and women’s empowerment, which is imperative for poverty alleviation and sustainable economic growth. At the individual level, financial empowerment provides the impetus for increasing women’s role in financial decision-making, and thus gender equality, in the household. At the community level, economically empowered women, by meeting their household well-being needs, contribute to eradicating poverty. At the macro level, women’s financial empowerment is critical in the long run for creating sustainable economic growth. These three levels interrelate through bilateral mechanisms to potentially form mutually reinforcing “virtuous spirals”. For example, women’s financial empowerment at the individual level may induce the upliftment of poorer communities which may, in turn, support and enable the empowerment of other women. It is therefore evident that, as aptly put by Postmus (2011a), financial literacy is essential for escaping all forms of abuse and poverty.

The concept of financial self-efficacy is derived from Bandura’s (2005) social cognitive theory which presumes that self-efficacy can be used to predict an individual’s domain-specific decisions and to anticipate their positive outcomes. “Self-efficacy” refers to “one’s belief in one’s ability to succeed in specific situations or accomplish a task” (Bandura, 1977). The theory of self-efficacy (Bandura, 1977) has received wide recognition in the social sciences and is used to explain self-efficacy in various fields or activities such as teaching self-efficacy, innovation self-efficacy, leadership self-efficacy, or financial self-efficacy. Financial efficacy has become an important concept in personal finance. For example, Danes and Haberman (2007) used financial self-efficacy to describe individuals’ ability to manage their finances in general. A number of studies examine the role of financial literacy in improving financial self-efficacy. For example, Mindra and Moya (2017) found the positive role of financial literacy in improving financial self-efficacy. In their important study, Noor et al. (2020) found a positive relationship between financial literacy and financial self-efficacy.

Coping behavior is the “cognitive and behavioral efforts made to master, tolerate, and reduce external and internal demands, and conflicts among them” (Folkman and Lazarus, 1980, 223). People use cognitive and behavioral skills to handle financial stress. There are two types of coping behaviors identified in personal finance: positive or active coping behavior (problem solving) and less positive or passive coping behavior (problem avoidance) (Cohen et al., 2008). Xiao et al. (2008) also identified two types of financial coping behaviors: proactive (saving and investing) and preventive (spending within a budget); we use the constructive of financial coping behavior as explained by Xiao et al. (2008). We believe that people who display proactive financial coping behavior regularly save money and deal with financial emergencies and financial stress in a better way. Whereas people with better preventive financial coping behavior have better budgeting and money management skills to keep detailed records of their money, and spending within the budget constraints.

A number of studies in the literature provide empirical evidence on the relationship between financial literacy and financial coping behavior. For instance, Sohn et al. (2012) hold that financial literacy develops financial coping behavior among individuals. Similarly, Bamforth et al. (2018) found financial literacy to be an important determinant of positive financial behaviors. Likewise, de Bassa Scheresberg (2013) also proposed that people with enhanced financial literacy have better financial behaviors for planning their retirement to improve financial well-being and saving money (financial coping behavior). Based on the above discussion, we propose the following hypotheses:

H1a: Financial literacy has a positive impact on financial empowerment.

H1b: Financial literacy has a positive impact on financial self-efficacy.

H1c: Financial literacy has a positive impact on financial coping behavior.

#### Financial Socialization, Financial Self-Efficacy, Financial Coping Behavior, and Financial Empowerment

The concept of financial socialization is evolved from Socialization theory (Moschis, 1987), which refers to learning different financial skills to improve financial well-being. Parents provide financial socialization opportunities by giving children pocket money, allowing them to pay their bills, and teaching them to spend and save money. The role of other socialization agents including teachers, relatives, friends, and the media is also important to develop financial socialization among children (Pinto et al., 2005). The role of parents is central among all financial socialization agents, as Pinto et al. (2005) found a negative association between parents’ financial communication and children’s financial difficulties. Gudmunson and Danes (2011) analyzed the literature on financial socialization between 1970 and 2010, and based on their analysis, they advocate the importance of family financial socialization in improving financial coping behavior and financial well-being among individuals in their later life. Similarly, Serido et al. (2010); Sohn et al. (2012) hold that financial socialization by parents and other social agents is significantly important in developing financial self-efficacy, financial coping behavior, and financial well-being among individuals. We, therefore, propose the following hypotheses:

H2a: Financial socialization has a positive impact on financial empowerment.

H2b: Financial socialization has a positive impact on financial self-efficacy.

H2c: Financial socialization has a positive impact on financial coping behavior.

#### Financial Self-Efficacy, Financial Coping Behavior, and Financial Empowerment

Financial efficacy is an influential determinant of financial behaviors like financial coping behavior and financial empowerment. Individuals who have a high level of financial self-efficacy, i.e., the confidence and ability to use finance in a better way, can practice proactive and preventive financial coping behaviors much better. Studies by Pearlin and Schooler (1978), Dietz et al. (2003), Lim et al. (2014), Serido et al. (2014), and Farrell et al. (2016) also support the proposition that higher levels of financial literacy enable individuals to manage their finances productively by adopting proactive and preventive financial coping behaviors.

Financial self-efficacy is anyone’s believed ability to manage his or her finances in a superior way. Several studies have noted a positive association between financial self-efficacy and objective and subjective financial well-being. For instance, Pearlin and Schooler (1978), Dietz et al. (2003), Perry and Morris (2005), and Lim et al. (2014) also conclude that greater financial self-efficacy leads to better financial management and well-being. Farrell et al. (2016) also investigated the relationship between higher levels of financial self-efficacy and improvements in financial wellbeing arising from an increase in savings and investment-related products and a decrease in debt-related products. In a recent study, Fosnacht and Calderone (2017) found that financial self-efficacy gives children the confidence to manage their finances in a better way and improve their financial well-being. We, therefore, propose the following hypotheses:

H3: Financial self-efficacy has a positive impact on financial coping behavior.

H4: Financial self-efficacy has a positive impact on financial empowerment.

#### Financial Coping Behavior and Financial Empowerment

As mentioned above, financial coping behavior refers to making cognitive and behavioral efforts to master and manage external and internal financial conflicts. Individuals with better financial coping behaviors reduce their financial stress and improve their financial empowerment. Although relatively little research has been done on the association between financial coping behavior and financial empowerment, several studies examined the role of financial coping behavior to improve financial well-being (Dyson and Renk, 2006; Cohen et al., 2008; Serido et al., 2010). In a recent study, Chakraborty and Abraham (2021) found a positive association between financial inclusion on social and financial empowerment. Based on this theoretical and empirical evidence, we present the following hypothesis:

H5: Financial coping behavior has a positive impact on financial empowerment.

#### The Conceptual Model

**FIGURE 1:**
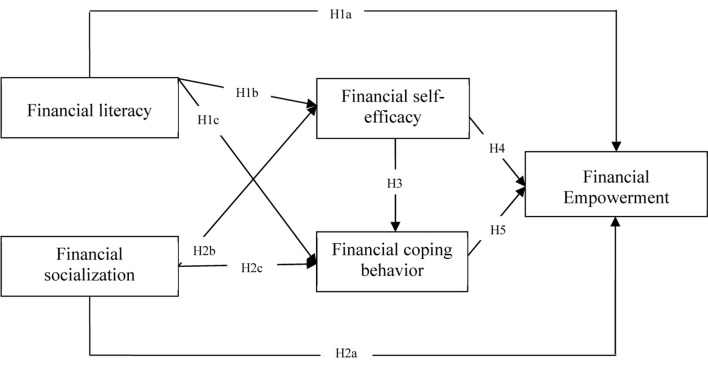
Conceptual model. All arrows indicate hypothesized positive relationships.

The conceptual model of this study is presented in Figure 1; the intendent variables include financial literacy and financial socialization that enhance financial self-efficacy and financial coping behavior, which in turn improve financial empowerment among Saudi women. Based on the premise of social cognitive theory (SCT), the current study assumes that learning through formal financial literacy education and training programs and interactions with financial socialization actors improve the financial attitudes and behaviors of Saudi women, which enhances their financial empowerment.

## Methodology

### Sample and Data Collection

This study examines the role of financial literacy and financial socialization in developing financial self-efficacy, financial coping behavior, and financial empowerment among Saudi women. The data were collected from Saudi women using self-administered, structured survey questionnaires by the research team and through online data collection platforms from across different cities of Saudi Arabia. Sample selection aimed to capture diverse socioeconomic backgrounds to enhance the generalizability of the findings. The survey questionnaire was translated into the Arabic language for the convenience of the respondents and to increase the response data. Since Saudi women are the population of this study, a population framework was not available, so we used a snowball sampling technique to collect data from different segments of our population. Many respondents were approached for data collection; however, 1,368 usable responses were received and used for data analysis in this study. This research is part of a project sponsored by the Deputyship of Research and Innovation, Ministry of Education in Saudi Arabia through the initiative of social sciences.

### Measures and Instruments

The instrument to measure financial empowerment is borrowed from Postmus et al. (2013). Postmus et al. (2013) adapted the original family empowerment scale (FES) developed by Koren et al. (1992). The sample items include “I feel confident in my ability to help myself grow and develop financially,” and “I can pretty much determine what will happen in my life, I can make my decisions,” and “It is important to be an earning hand in order to get power in decision-making and respect in the family and society.” The instrument consists of six items, measured on a five-point Likert scale where 1 is strongly disagree and 5 is strongly agree.

### Data Analysis

In this study, the analysis of data comprises the evaluation of the financial empowerment model and the relevant hypotheses were performed using partial least squares-structure equation modeling (PLS-SEM) or PLS path modeling (Sarstedt et al., 2016; Hair et al., 2017). PLS-SEM and its applications are widely used as a relevant statistical technique in entrepreneurship research. Based on the updated recommendations and following relevant studies in entrepreneurship research (Ali et al., 2019; Lin et al., 2021), this study applied PLS-SEM as a statistical technique to analyze the data. It is capable of simultaneously analyzing the measurement and structure models of financial empowerment. Briefly, the analysis of PLS-SEM comprises two sub-models: (1) a measurement model which examines the reliability and validity of the measurement model (standardized factor loadings, Cronbach’s alpha, Dijkstra–Henseler’s rho, composite reliability, average variance extracted, and discriminant validity), (2) a structural model which examines the associations among independent and dependent constructs including collinearity concern, predictive capability (*R*^2^), predictive relevance (*Q*^2^), and testing hypothesis. SmartPLS 3.3.3 software (Ringle et al., 2015) is used to estimate the research model.

### Measurement Model

The assessment of the measurement model depends on the reliability and validity of the measurement model which comprises the assessment of standardized factor loadings, Cronbach’s alpha, Dijkstra–Henseler’s rho, composite reliability, average variance extracted, and discriminant validity. A generally accepted rule of thumb is to accept an item with a standardized factor loading of 0.70 (Fornell and Larcker, 1981) with a two-tailed *p* ≤ 0.05, while a standardized factor loading of 0.50 (*p* ≤ 0.05, two-tailed test) is considered practically significant for exploratory research. The results of standardized factor loadings, standard errors, and *t*-values are provided in Table 1. All standardized factor loadings are above 0.50 and are statistically significant (*p* = 0.05, two-tailed), suggesting the reliability of each item. Construct reliability represents how much of the variation in an item is explained by the construct and is referred to as the variance extracted from the item. Construct reliability is estimated for each construct using: (1) Cronbach’s alpha (α), (2) Dijkstra–Henseler’s rho (ρA), and (3) composite reliability (CR). In all three cases, a value of reliability ≥0.70 is deemed significant. The values of α, ρA, and CR of each construct are above 0.70, suggesting acceptable construct reliability of each construct in the measurement model. In general, AVE refers to the sum of variance a construct gains from its associated items with relation to the measurement variance and its value should be greater than 0.50 (Fornell and Larcker, 1981). The AVE of each construct is provided in Table 1. All the AVE values pass the minimum threshold level for significance, suggesting that each construct has high convergent validity in the measurement model.

**TABLE 1:** Measurement model results: loadings, construct reliability, and convergent validity coefficients.

Construct	Code	Loading^*a*^	SE	*t*-value	α	ρ_*A*_	CR	AVE^*b*^
*Financial literacy*					0.92	0.93	0.93	0.62
	FL1	0.67	0.02	36.26				
	FL2	0.77	0.01	60.43				
	FL3	0.68	0.02	35.70				
	FL4	0.77	0.01	64.88				
	FL5	0.77	0.01	61.58				
	FL6	0.82	0.01	94.84				
	FL7	0.79	0.01	68.69				
	FL8	0.82	0.01	64.63				
	FL9	0.95	0.00	99.89				
*Financial socialization*					0.90	0.91	0.92	0.56
	FS1	0.77	0.01	60.47				
	FS2	0.70	0.02	36.01				
	FS3	0.65	0.02	30.91				
	FS4	0.81	0.01	67.31				
	FS5	0.80	0.01	76.02				
	FS6	0.79	0.01	63.34				
	FS7	0.74	0.01	52.30				
	FS8	0.79	0.01	64.28				
	FS9	0.67	0.02	42.31				
*Financial self-efficacy*					0.77	0.79	0.84	0.50
	FSE1	0.57	0.03	20.46				
	FSE2	0.52	0.03	17.68				
	FSE3	0.74	0.02	47.61				
	FSE4	0.76	0.02	49.52				
	FSE5	0.70	0.02	32.91				
	FSE6	0.78	0.01	58.54				
*Financial coping behavior*					0.95	0.95	0.96	0.80
	FCB1	0.86	0.01	99.02				
	FCB2	0.88	0.01	109.29				
	FCB3	0.90	0.01	146.76				
	FCB4	0.86	0.01	86.54				
	FCB5	0.94	0.00	112.51				
	FCB6	0.95	0.00	157.30				
*Financial empowerment*					0.84	0.85	0.88	0.56
	FE1	0.67	0.02	28.42				
	FE2	0.72	0.02	40.30				
	FE3	0.75	0.02	46.73				
	FE4	0.74	0.02	47.29				
	FE5	0.80	0.01	75.18				
	FE6	0.81	0.01	84.32				

*^*a*^All loadings are significant at *p* < 0.001 – [based on *t*_(499)_, two-tailed test]. SE, standard error; α, Cronbach’s alpha; CR, composite reliability; ρ_*A*_, Dijkstra–Henseler’s rho; AVE, average variance extracted.*

*^*b*^Percentage of variance of item explained by the construct.*

Discriminant validity is computed by assessing (1) Fornell–Larcker criterion and (2) the heterotrait-monotrait (HTMT) approach. The Fornell–Larcker criterion compares the square root of AVE with the correlations among constructs. The values of AVE are provided below the diagonal in Table 2, indicating that for each construct, the square root of its AVE is higher than its correlation with any other construct. The values of HTMT are significantly below 0.85 as in the range of acceptable minimum threshold (Henseler et al., 2015), suggesting discriminant validity of all constructs in the measurement model.

**TABLE 2:** Mean, standard deviations, correlations, and discriminant validity results.

	Mean	SD	VIF	1	2	3	4	5
1. Financial literacy	NA	NA	1.28	*0.79*	0.67	0.62	0.50	0.63
2. Financial socialization	3.28	0.79	1.13	0.63	*0.75*	0.65	0.53	0.70
3. Financial self-efficacy	3.57	0.69	1.42	0.53	0.55	*0.69*	0.51	0.77
4. Financial coping behavior	3.39	0.88	1.32	0.48	0.51	0.44	*0.90*	0.50
5. Financial empowerment	3.50	0.71	1.66	0.57	0.63	0.63	0.45	*0.75*

*SD, standard deviation; VIF, variance inflation factor. Correlation is significant at the 0.05 level (two-tailed). Diagonal and italicized elements are the square roots of the AVE (average variance extracted). Below the diagonal elements are the correlations between the construct’s values. Above the diagonal elements are the HTMT values.*

### Structural Model

The validation of a structural model is assessed through evaluation of collinearity in the structural model, predictive capability (*R*^2^), predictive relevance (*Q*^2^), and testing hypothesis. First, to treat biasness in the path coefficient model, the multicollinearity in the structural model is examined. The multicollinearity is examined through the analysis of variance inflation factor (VIF) approach. The values of VIF are provided in Table 2. All VIF values are below 3.00 (Hair et al., 2017), suggesting that multicollinearity is not a concern in the structural model. The predictive capability of the structural model is assessed through the coefficient of determination (or *R*^2^ value) of all dependent constructs in the structural model. The two independent constructs, which are financial literacy and financial socialization, collectively explain financial self-efficacy (36%). The three independent constructs, which are financial literacy, financial socialization, and financial self-efficacy, collectively explain financial self-efficacy (32%). Finally, the four independent constructs, which are financial literacy, financial socialization, financial self-efficacy, and financial coping behavior, collectively explain financial empowerment (53%) as shown in Table 3. Furthermore, the result of the predictive capability of the structural model is further supported through the assessment of the predictive relevance *Q*^2^ value. The predictive relevance *Q*^2^ value is commonly measured through the blindfolding method. With an omission distance of 7 for every dependent construct, the blindfolding procedure yielded the Stone-Geisser-Criterion *Q*^2^ value, which represents the cross-validated redundancy for dependent constructs (Hair et al., 2017). The predictive relevance *Q*^2^ values are provided in Table 3, which are well above zero (Hair et al., 2017), suggesting the confirmation of predictive relevance of the structural model.

**TABLE 3:** Determination coefficients (*R*^2^) and predictive relevance (*Q*^2^) of endogenous (omission distance **=** 7).

Constructs	*R*^2^ values	Threshold	*Q*^2^ values	Threshold
Financial self-efficacy	0.36	≥0.33 (moderate)	0.17	
Financial coping behavior	0.32	≥0.67 (substantial)	0.25	>0
Financial empowerment	0.53	≥0.33 (moderate)	0.30	

To examine the relevance and significance of each hypothesis, this study utilized the bootstrapping technique. Table 4 reports the results of statistically significant hypotheses which are obtained through using the bootstrapping technique (1,368 responses, 5,000 samples with no sign change option). Following Roldán and Sánchez-Franco (2012), *t*-statistic with one-tailed test and 95% bias-corrected (BCa) confidence interval (CI) are used to confirm the significance of the hypotheses (Hair et al., 2017). The results of the testing of the hypothesis suggest that all the proposed hypotheses are accepted (Table 4). In the line with previous studies, the statistical results of PLS-SEM suggest that financial literacy has a positive and significant impact on financial empowerment (H1a; *β* = 0.16; *p* < 0.01), financial self-efficacy (H1b; *β* = 0.30; *p* < 0.01), and financial coping behavior (H1c; *β* = 0.21; *p* < 0.01), thus supporting H1a, H1b, and H1c. Financial socialization has a positive and significant impact on financial empowerment (H2a; *β* = 0.31; *p* < 0.01), financial self-efficacy (H2b; *β* = 0.36; *p* < 0.01), and financial coping behavior (H2c; *β* = 0.27; *p* < 0.01), thus H2a, H2b, and H2c are accepted. Financial self-efficacy has a positive and significant impact on financial coping behavior (H3; *β* = 0.18; *p* < 0.01) and financial empowerment (H4; *β* = 0.35; *p* < 0.01), thus H3 and H4 are accepted. Finally, financial coping behavior has a positive and significant impact on financial empowerment (H5; *β* = 0.10; *p* < 0.01), thus H5 is accepted. Furthermore, the value of the standardized root-mean-square residual (SRMR) is 0.07, satisfying that the threshold limit should be less than 0.08, while a zero value suggests a perfect model fit. In this study, the index of SRMR confirmed the overall goodness-of-fit measure for validation of the structural model (Hair et al., 2017).

**TABLE 4:** Significant testing results of the structural model path coefficients.

Structural path	Standardized path coefficient	*t*-value	Significant difference (*p* < 0.05)?	95% BCa confidence interval		Conclusion
				Low	High	
Financial literacy → Financial empowerment	0.16***	5.53	Yes	0.11	0.20	H1a; Accepted
Financial literacy → Financial self-efficacy	0.30***	11.19	Yes	0.26	0.34	H1b; Accepted
Financial literacy → Financial coping behavior	0.21***	6.84	Yes	0.16	0.26	H1c; Accepted
Financial socialization → Financial empowerment	0.31***	11.52	Yes	0.27	0.36	H2a; Accepted
Financial socialization → Financial self-efficacy	0.36***	12.41	Yes	0.31	0.41	H2b; Accepted
Financial socialization → Financial coping behavior	0.27***	7.84	Yes	0.22	0.33	H2c; Accepted
Financial self-efficacy → Financial coping behavior	0.18***	6.17	Yes	0.13	0.23	H3; Accepted
Financial self-efficacy → Financial empowerment	0.35***	14.04	Yes	0.30	0.39	H4; Accepted
Financial coping behavior → Financial empowerment	0.06**	2.79	Yes	0.03	0.10	H5; Accepted

*ns, non-significant; *t* (0.05, 4999) = 1.645; *t* (0.01, 4999) = 2.327; *t* (0.001, 4999) = 3.092.*

**p < 0.01; ***p < 0.001, based on *t* (4999), one-tailed test.

*BCa, bias-corrected confidence interval. Bootstrapping based on *n* = 5000 subsamples.*

## Discussion and Conclusion

The objective of this study includes examining the role of financial literacy and financial socialization in developing financial self-efficacy, financial coping behavior, and improving financial empowerment among women in Saudi Arabia. The data are collected from different women respondents who belong to different segments of Saudi society. We employed the PLS path modeling technique to test the hypotheses proposed in the conceptual model. The study found a positive role of financial literacy in developing financial self-efficacy, financial coping behavior, and financial empowerment. Similarly, financial socialization is also positively related to the development of financial self-efficacy, financial coping behavior, and financial empowerment. Likewise, financial self-efficacy and financial coping behavior are positively related to financial empowerment.

All hypotheses initially proposed are accepted in this study, which explains the context of two important specific contributions of this study. First, the role of financial literacy is very important to improve the financial empowerment among Saudi women, particularly in the context of Saudi society, which is traditionally male-dominated, and the socio-cultural values do not allow women to participate in financial activities. Second, financial socialization of women is imperative with different social actors including parents, teachers, and other family members. This stresses the importance of financial parenting of children, particularly girls who will later transfer these values to future generations. Although Saudi Arabia is traditionally a male-dominated society that derives its values from religion, where men are supposed to be responsible for economic activities and women are supposed to rear children and manage the household, the fact remains that Saudi society is transforming rapidly, and the government has introduced a number of well-planned interventions and initiatives to improve women’s participation in the labor market and other walks of life. As a result, Saudi women are excited to play important roles in the socioeconomic development of the Kingdom. Although the Saudi government has initiated some financial literacy programs, they are not sufficient given the size of the population and geographical landscape of the Kingdom. Moreover, these programs are generally designed for Saudi youth, whereas financial literacy is important for all segments of Saudi women, particularly those who are less educated and living in rural areas. Clearly, there is a dire need to assess the financial literacy among different segments of Saudi women and propose evidence-based policy measures to promote financial literacy, to improve financial well-being, and economic empowerment of Saudi women. We therefore propose that the vision to increase women’s active participation in socio-economic spheres of life and their financial empowerment can only be achieved by provision of high levels of financial literacy and training, and financial socialization so that they make effective decisions to improve their financial well-being in their personal and economic lives.

The findings of this study reinforce the assumptions of the social cognitive theory, where external factors like financial literacy development institutions and financial socialization factors are co-interrelated with Saudi women in improving their financial management behavior and financial empowerment. Thomas and Gupta (2021) also endorsed SCT by examining the relationship between financial literacy development factors, individuals, and their attitude toward financial well-being.

The findings of this study are consistent with many previous studies, for instance, we found a positive association between financial literacy, financial self-efficacy, and financial empowerment. Scholars like Lusardi and Mitchell (2007a,b, 2008, 2011), Lusardi and Tufano(2010, 2009), Postmus (2010, 2011a, 2011b), Kim and Chatterjee (2013); Ali et al. (2015), Karakurum-Ozdemir et al. (2019), and Goncalves et al. (2021) believe that financial literacy is an important determinant of financial well-being and financial empowerment.

### Theoretical Implications

Philippas and Avdoulas (2020) and Goncalves et al. (2021) highlight the gendered gap in financial literacy and financial well-being in the literature. Although there is some research available on financial literacy among women in developed countries, there exists sparse evidence on financial literacy and financial empowerment in developing countries and particularly in traditionally male-dominated and religious societies like Saudi Arabia, where men are the breadwinners and are responsible for handling finances and the related decision-making. Research on financial literacy, financial socialization, and financial empowerment is much warranted to document empirical evidence, offer theoretical frameworks, and propose policy recommendations.

### Practical Implications

The practical implication of this study includes an increase in interventions to promote formal financial literacy education and training across the Kingdom and different segments of women ranging from students to homemakers to working women to yield better outcomes of such programs. Although there exist some programs in Saudi Arabia to promote financial literacy, they need to scale up and more rigorous programs need to be introduced. Financial literacy should be part of the curriculum at different educational levels and across different disciplines of education. Financial socialization actors should be more active to act as role models for women of different age groups and occupations to inspire them to develop their financial management skills to yield financial well-being and empowerment. The role of parents is of paramount importance in this regard. Financial parenting should be promoted in the Kingdom to train young women to develop sound financial literacy and financial management skills that will increase their financial well-being and empowerment. Similarly, teachers, society, and social media should also promote a learning attitude toward the development of financial literacy and financial management skills to spend their financial life empowered.

Despite the vital role of financial literacy in creating financial empowerment, several challenges and limitations need to be addressed. Among the most pressing challenges are the low levels of basic literacy and financial inclusion in poorer communities (Mayoux, 2002), especially in developing countries. Additionally, societal and cultural barriers that inhibit women from exercising control over even “female-specific” assets such as jewelry or creating and accessing financial support networks (Mayoux, 2002) present significant barriers to the proliferation of financial empowerment. Even if these obstacles are overcome, the assumption that financial education will induce individuals to improve their financial behaviors may not hold true (Williams, 2007). Also, financial literacy initiatives may not produce the desired financial empowerment effects if they are designed solely to “responsibilize” consumers without sufficient reforms in financial firms and regulatory mechanisms (Williams, 2007). If developed in this manner, financial literacy initiatives will be no more than tools for increasing financial firms’ revenues and relieving regulators from their duty of protecting the interests of the general public. Rather than empowering individuals, such initiatives may instead subordinate them to exploitative firms.

Therefore, financial literacy initiatives aimed at empowering and improving the well-being of women should adopt a “strategic gender justice” approach, as recommended by Mayoux (2002). Under this approach, financial literacy is just one part of a larger, strategic initiative that includes mainstreaming empowerment in core financial activities, identifying and developing financial products that support empowerment (especially microfinance products) through participatory market research, providing non-financial services to enhance the penetration and impact of empowerment initiatives, and developing networks through client participation (Mayoux, 2002). Naturally, this would require the concerted effort and long-term commitment of various stakeholders including regulators, financial firms, and communities.

### Limitations and Directions for Future Research

Like other studies, this study also suffers from certain limitations; the findings of this study are subject to the data obtained from the respondents in research and may not be generalized to a larger population. The findings are based on self-reported data that could entail some self-servicing biases by some respondents. We suggest the use of other variables to predict the outcomes of financial literacy and financial socialization; similarly, different variables can also be used to better predict the antecedents of financial well-being. We have assessed women’s knowledge of basic financial literacy concepts; an examination of medium and advance levels of financial literacy concepts can yield more interesting outcomes. Also, we have used a single construct of financial socialization that includes multiple actors. We suggest that each socialization factor should be examined in detail on the role of different factors in developing Saudi women’s financial management skills and financial empowerment. Further, we have collected cross-sectional data, whereas future research can use a longitudinal research design to yield more confident outcomes.

## Data Availability Statement

The datasets presented in this article are not readily available because this is a funded research project and data can only be shared subject to the approval of the funding agency. Requests to access the datasets should be directed to IA, imranalinim@gmail.com.

## Author Contributions

IA and YS contributed to the theory development and writing of the manuscript. SB contributed to the data analysis and write-up. MA contributed with data analysis and write-up of the interpretations of the results. All authors contributed to the article and approved the submitted version.

## Conflict of Interest

The authors declare that the research was conducted in the absence of any commercial or financial relationships that could be construed as a potential conflict of interest.

## Publisher’s Note

All claims expressed in this article are solely those of the authors and do not necessarily represent those of their affiliated organizations, or those of the publisher, the editors and the reviewers. Any product that may be evaluated in this article, or claim that may be made by its manufacturer, is not guaranteed or endorsed by the publisher.
